# Unraveling the Genetic and Molecular Architecture of Autism Spectrum Disorder: Implications for Clinical Genetics and Genomic Diagnostics

**DOI:** 10.3390/ijms27073278

**Published:** 2026-04-04

**Authors:** Simone Treccarichi, Mirella Vinci, Miriam Virgillito, Antonino Musumeci, Francesca Bruno, Carla Papa, Rosanna Galati Rando, Pietro Marano, Donatella Greco, Antonio Fallea, Desiree Brancato, Siria Calì, Gresheen Garcia, Concetta Federico, Salvatore Saccone, Francesco Calì

**Affiliations:** 1 Oasi Research Institute-IRCCS, 94018 Troina, Italy; streccarichi@oasi.en.it (S.T.); mvinci@oasi.en.it (M.V.); amusumeci@oasi.en.it (A.M.); cpapa@oasi.en.it (C.P.); rgalati@oasi.en.it (R.G.R.); pmarano@oasi.en.it (P.M.); dgreco@oasi.en.it (D.G.); afallea@oasi.en.it (A.F.); cali@oasi.en.it (F.C.); 2 Department of Biological, Geological and Environmental Sciences, University of Catania, 95124 Catania, Italy; virgillito.miriam@studium.unict.it (M.V.); desiree.brancato@phd.unict.it (D.B.); siria.cali@studium.unict.it (S.C.); concetta.federico@unict.it (C.F.); 3 Department of Medicine and Surgery, Kore University of Enna, 94100 Enna, Italy; francesca.bruno@unikore.it; 4 Department of Agriculture, Food and Environment (Di3A), University of Catania, 95131 Catania, Italy; gresheen.garcia@phd.unict.it

**Keywords:** neurodevelopmental disorder, diagnostic yield, array-CGH, WES, WGS, chromosomal abnormalities, WGS, CNVs, SNVs

## Abstract

Autism spectrum disorder (ASD) is a neurodevelopmental condition that occurs in early childhood, characterized by a broad range of clinical manifestations and impairments in social communication. It represents one of the most prevalent neurodevelopmental disorders, affecting approximately 1% of the general population. The phenotypic heterogeneity of ASD arises from different genetic causes, including chromosomal abnormalities, copy number variants (CNVs), and single-nucleotide variants (SNVs), which may occur as de novo or inherited events. Moreover, the polygenic and multifactorial nature of ASD, together with epigenetic regulation and environmental influences, contributes substantially to its complex genetic architecture. Molecular diagnosis remains challenging and relies on multiple genomic approaches, such as array comparative genomic hybridization (array-CGH), whole-exome sequencing (WES), and whole-genome sequencing (WGS); however, the diagnostic yields of these methods remain limited, reflecting the complexity of ASD’s genetic architecture. Notably, ASD-associated genes converge on key biological pathways, particularly those involved in transcriptional regulation, chromatin remodeling, synaptic function, and neuronal signaling. These include well-established risk genes such as *CHD8*, *ADNP*, *ARID1B*, *SHANK3*, *SYNGAP1*, *SCN2A*, *GRIN2B*, *FOXP1*, and *DYRK1A*, among others. This review summarizes the current knowledge on the genetic basis of ASD, highlighting key aspects of its complex genetic architecture. By integrating evidence from major clinical and research databases, it provides a clearer understanding of the underlying mechanisms, supporting improved diagnosis and future research and therapeutic strategies.

## 1. Introduction

According to the *Diagnostic and Statistical Manual of Mental Disorders, Fifth Edition* (DSM-5), autism spectrum disorder (ASD) is a neurodevelopmental disorder characterized by persistent deficits in social communication and social interaction, accompanied by restricted, repetitive patterns of behavior, interests, or activities [1]. Core features include impairments in social–emotional reciprocity, atypical use of nonverbal communicative behaviors, and difficulties in developing, maintaining, and understanding interpersonal relationships. Individuals with ASD frequently exhibit stereotyped or repetitive motor movements, rigid adherence to routines, and ritualized patterns of behavior [2]. As previously outlined, ASD exhibits an estimated heritability of 70–90%, reflecting the substantial contribution of genetic factors to familial transmission [3,4].

Diagnosis of ASD involves analyzing the child’s developmental history and monitoring behavioral and cognitive features, which must conform to the expert-defined criteria outlined in the DSM-5. The complexity of diagnosing ASD arises from the heterogeneity of phenotypic presentations and the variable severity observed across patients. Although the DSM-5 provides standardized diagnostic criteria, it presents certain limitations, including incomplete coverage of behaviors and symptoms characteristic of autism, challenges in identifying individuals with mild manifestations, and difficulties for clinicians in determining the relevance of specific features, such as motor delays, due to insufficient guidance for differentiating core autistic traits from unrelated developmental variations [5]. The diagnosis of autism relies on an integrative approach that includes a thorough review of the patient’s clinical history, multiple structured interviews with caregivers, and behavioral observations using standardized tools such as the Autism Diagnostic Observation Schedule (ADOS) and clinical interview instruments, including the Developmental, Dimensional and Diagnostic Interview (3Di) or the Autism Diagnostic Interview Revised (ADI-R), and genetic evidence [6]. An early diagnosis is key to enabling the best clinical approach. Several studies support the benefits of early diagnosis, as it allows for timely intervention that can improve cognitive, language, and social–emotional functioning [7]. ASD encompasses clinically and genetically heterogeneous conditions that are commonly classified as syndromic, non-syndromic, or idiopathic [8,9]. Syndromic ASD refers to cases in which autistic features occur as part of a defined genetic or chromosomal syndrome, often accompanied by additional neurological or systemic manifestations, such as intellectual disability, dysmorphic features, or epilepsy. Non-syndromic ASD describes individuals who meet the diagnostic criteria for ASD in the absence of a recognizable syndrome or major congenital anomalies, although pathogenic genetic variants may still be identified. In contrast, idiopathic ASD refers to cases for which no single causative genetic or environmental factor has been identified, reflecting the complex and multifactorial nature of ASD etiology involving polygenic risk and environmental modifiers.

Pioneering studies on multiplex families and twin cohorts have demonstrated a strong heritable component of ASD; however, identifying a single causative genetic variant has proven challenging [10]. Consequently, a growing body of evidence supports the view that autism arises from the combined effects of multiple genetic variants, consistent with a polygenic model of risk [11]. The Simons Foundation Autism Research Initiative (SFARI) aims to systematically collect and curate genetic data on genes potentially associated with ASD [12]. It maintains the SFARI Gene database, a regularly updated, expert-curated resource that integrates evidence from multiple study types, including sequencing analyses, copy number variant studies, and genome-wide association studies. This database serves as a comprehensive and evolving reference for ASD candidate genes, and its Human Gene module currently includes more than 1200 genes (database updated on 14 January 2026) with reported or suspected links to ASD. SFARI Gene uses a structured evidence-based scoring system to classify genes according to the strength of their association with ASD. Each gene is assigned to a category that reflects the quantity and quality of supporting data. The principal categories are:

- Syndromic—genes in which pathogenic variants are associated with ASD in the context of a broader, well-defined genetic syndrome, typically accompanied by additional clinical features beyond the core ASD criteria.
- High confidence—genes with strong and replicated evidence for ASD association, often supported by multiple independent de novo protein-disrupting variants.
- Strong candidate—genes supported by moderate genetic evidence, such as at least two likely gene-disrupting de novo variants, or robust and replicated association signals with functional support.
- Suggestive evidence—genes with preliminary or limited support, including single de novo disruptive variants, unreplicated association findings, or rare inherited variants lacking rigorous case–control statistical validation.

This tiered classification framework supports both research prioritization and variant interpretation by providing a transparent estimate of gene–ASD association strength and facilitating ongoing reclassification as new data emerge.

Several types of genetic mutations have been identified as contributing to the onset of ASD, including chromosomal abnormalities, copy number variants (CNVs), and single-nucleotide variants (SNVs). These genetic alterations may arise de novo or be inherited from the parents [11]. Genome-wide association studies (GWASs) have made a major contribution to the identification of hundreds of DNA polymorphisms clustered within genetic loci associated with ASD [13]. However, these studies are primarily designed for research purposes and are not routinely used for molecular diagnosis.

In addition to large-scale genomic studies, recent gene-centered investigations have identified novel candidate genes involved in neurodevelopmental processes, including neuronal adhesion, synaptic organization, and intracellular signaling, further supporting the contribution of rare variants to ASD heterogeneity [14,15,16].

### 1.1. Objective and Research Question

This literature review aims to provide a comprehensive and up-to-date overview of the major genetic alterations implicated in ASD. Specifically, this work seeks to synthesize the current evidence on key genetic variants, ASD-associated genes, and the molecular pathways involved in disease pathogenesis. In addition, this work discusses the strengths and limitations of the current genetic diagnostic approaches and highlights emerging mechanisms that may contribute to ASD. By integrating findings from recent genomic and functional studies, this review aims to improve the understanding of ASD biology and support future research and diagnostic strategies. We would like to emphasize that the present review is guided by the following research questions: to what extent do the diverse genetic alterations associated with ASD converge on shared biological pathways, and how can this knowledge improve our understanding of disease pathogenesis and diagnostic strategies? By addressing these questions, this work aims to move beyond a purely descriptive overview and provide an integrated perspective on the molecular mechanisms underlying ASD.

### 1.2. Searching Strategy

This review was conducted through a structured, but non-systematic, search of the literature to provide an updated overview of the genetic basis of ASD. Bibliographic databases including PubMed, Scopus, and Web of Science were queried using combinations of keywords related to ASD genetics, CNVs and relevant molecular pathways. Additional studies were identified through the manual screening of reference lists of selected articles. Given the narrative nature of this review, no formal inclusion or exclusion criteria or systematic screening protocols (e.g., PRISMA guidelines) were applied. Instead, priority was given to peer-reviewed studies of high relevance, including recent large-scale genomic analyses, functional studies, and key review articles, to provide a comprehensive and balanced synthesis of the current knowledge. Several databases, including the OMIM (https://www.omim.org/) (accessed on 16 February 2026), MalaCards (https://www.malacards.org/) (accessed on 16 February 2026), and SFARI (https://gene.sfari.org/) (accessed on 16 February 2026), were queried to identify ASD-related genes.

## 2. Genetic Counseling and Genetic Testing for ASD Diagnosis

Despite the complex genetic architecture of ASD, research aimed at identifying novel genes and pathogenic variants associated with the condition is rapidly evolving. As genetic testing becomes increasingly integrated into clinical practice, the interpretation and communication of results to families of affected individuals require specialized expertise in ASD genetics [17,18]. Proper result disclosure demands careful consideration of variant pathogenicity, uncertainty, and clinical relevance.

**Figure 1:**
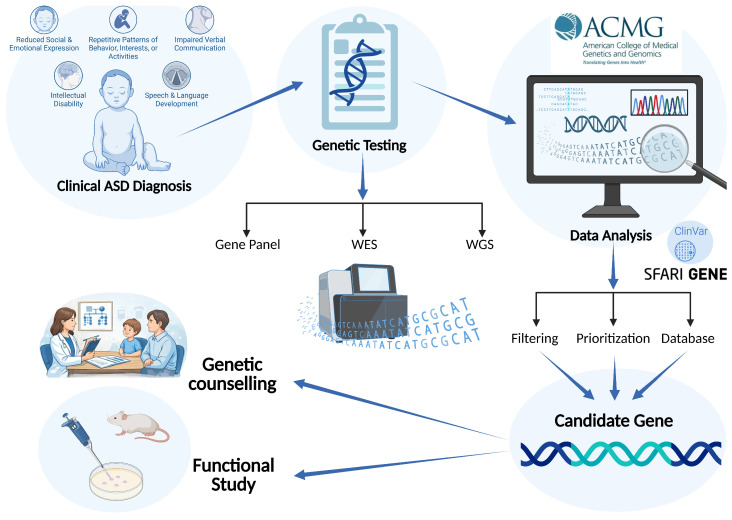
The integrated workflow for the genetic diagnosis and clinical interpretation of ASD. A schematic overview of the clinical and genomic pipeline used to investigate the genetic architecture of ASD. The process begins with clinical ASD diagnosis based on neurodevelopmental features, including reduced social interaction, repetitive behaviors, language impairment, and intellectual disability. Following clinical assessment, patients undergo genetic testing approaches, such as targeted gene panels, whole-exome sequencing (WES), or WGS. Variant interpretation is performed through bioinformatics data analysis, including filtering, prioritization, and database mining using curated genomic resources (e.g., OMIM, ClinVar, SFARI Gene, and ACMG guidelines). Candidate genes identified through this process may be further evaluated through functional studies to assess their biological relevance. The results are subsequently integrated into genetic counseling, supporting clinical decision making, risk assessment, and personalized management strategies. The arrows indicate the iterative nature of the workflow, highlighting the feedback loop between functional validation, genetic interpretation, and clinical care.

Given this complexity, genetic testing and counseling in ASD raise important ethical and practical challenges, including the management of variants of uncertain significance, incidental findings, limitations of predictive value, and the potential psychosocial impact on families. These considerations highlight the need for experienced multidisciplinary teams and well-defined counseling frameworks to ensure the responsible and clinically meaningful use of genetic information. Recent studies have further emphasized the importance of integrating genomic findings with clinical phenotyping to improve diagnostic interpretation and counseling outcomes, particularly in heterogeneous neurodevelopmental disorders [19,20]. Genetic tests have been gaining prominence in recent years. The recurrence risk is increased in families with a child with autism, estimated at 8.4-fold for siblings and 2-fold for cousins, with heritability estimated at 65–90%, reflecting the important role of genetics in the etiology of ASD [21]. Furthermore, genetic evaluation is recommended by the American College of Medical Genetics and Genomics (ACMG), which provides guidelines for selecting the most appropriate diagnostic test based on a patient’s clinical features [22]. Genetic tests are used to detect de novo or inherited variants, as well as rare or common variants. These variants can be classified by type, including chromosomal abnormalities, CNVs, and SNVs (missense, nonsense, frameshift). In this context, next-generation sequencing (NGS)-based diagnostic pipelines have become essential tools in clinical genetics, enabling the systematic identification and prioritization of candidate variants across diverse classes of genomic alterations [19]. The integrated diagnostic workflow, spanning clinical ASD evaluation, genomic testing, bioinformatic interpretation, and genetic counseling, is schematically summarized in Figure 1.

### Genetic Testing Approaches in ASD

Several studies have highlighted the variable diagnostic yields of different genetic testing approaches in ASD. Chromosomal microarray analysis (CMA) shows a diagnostic yield ranging from approximately 9% to 20% [23,24,25], primarily through the identification of pathogenic CNVs associated with ASD etiology. In clinical practice, CMA is traditionally recommended as a first-tier test for detecting CNVs, particularly in patients with syndromic features or congenital anomalies [26]; however, it is limited in identifying single-nucleotide variants and small insertions/deletions. In parallel, numerous studies have evaluated the feasibility of whole-exome sequencing (WES) as a diagnostic tool in ASD, reporting diagnostic yields of approximately 10–30% [24,27,28]. Importantly, the diagnostic yield of WES may increase to around 30% when broader phenotypic spectra are considered, such as cohorts including multiple neurodevelopmental disorders (NDDs) rather than ASD alone [29], or when WES is applied within an integrated diagnostic framework that combines sequencing with CMA [30]. Consistent with these findings, recent clinical studies have demonstrated that implementing WES as a first-line diagnostic approach in neurodevelopmental disorders can significantly enhance variant detection and clinical interpretation, particularly when supported by detailed phenotypic characterization [31]. WES, in fact, has emerged as a highly effective first-tier diagnostic tool for individuals with unexplained NDDs, as it not only improves diagnostic resolution but also has a significant impact on clinical management [32]. The observed variability in diagnostic yields is influenced by several factors, including the study design, variant interpretation criteria, and the genetic background of the tested population. Notably, population consanguinity plays a significant role in diagnostic success. In highly consanguineous populations, WES—particularly trio-based approaches—has been shown to yield significantly higher diagnostic rates [33]. Trio-based WES facilitates the identification of de novo variants, which are considered a major contributor to ASD etiology, as well as recessive variants enriched in consanguineous cohorts. Together, these findings highlight the importance of tailoring genetic testing strategies to both clinical presentation and population-specific genetic characteristics to maximize diagnostic yields. In contrast, WGS demonstrates a higher diagnostic yield, ranging from approximately 20% to 40% [34,35]. Notably, when WGS is applied to broader cohorts encompassing multiple NDDs, the diagnostic yield increases to around 40% [36]. It is important to note that many WGS-based studies have been conducted on heterogeneous NDD populations rather than on ASD-specific cohorts, which may partially account for the higher diagnostic yields reported compared with studies focused exclusively on ASD. Notably, its routine clinical use is currently limited by cost and data interpretation challenges.

While WES is currently a widely available and routinely performed diagnostic tool, a substantial proportion of patients remain molecularly undiagnosed. These unresolved cases underscore the need for complementary approaches, including WGS, functional validation studies, and integrated genetic diagnostic strategies, to identify non-coding variants, structural rearrangements, regulatory elements, and complex gene–gene interactions that are not detectable by WES alone. Despite their currently limited diagnostic yield, targeted gene panels have also been employed as diagnostic tools [37]. Currently, many authors recognize that an integrated genetic approach represents the most effective strategy for both ASD diagnosis and research. However, limitations in infrastructure, access to next-generation sequencing technologies, and resource availability often restrict its practical implementation.

Recent evidence indicates that long-read genome sequencing (lrGS) technologies provide more accurate and comprehensive variant detection than short-read genome sequencing (srGS), including in neurodevelopmental disorders [38]. However, the magnitude of improvement in molecular diagnostic yields for rare diseases has not yet been fully quantified. Notably, lrGS enables the identification and characterization of complex genomic rearrangements (CGRs), including large duplications that have been shown to segregate with ASD in multiple affected families [39]. In addition to other molecular technologies used in the clinical setting, lrGS enables direct haplotype phasing, allowing for the identification of groups of genetic variants that are inherited together on the same parental chromosome due to genetic linkage [40]. Table 1 summarizes the main NGS approaches currently used for the genetic diagnosis of ASD.

## 3. Genetic Architecture of ASD

### 3.1. Chromosomal Abnormalities and Copy Number Variants (CNVs)

Historically, karyotyping represented the first-line genetic test for patients with suspected ASD, as technologies capable of detecting copy number variants (CNVs) and single-nucleotide variants (SNVs) were not yet available [11]. Chromosomal abnormalities comprise whole-chromosome aneuploidies or partial chromosomal alterations. Several lines of evidence indicate an increased risk of developing ASD in individuals with chromosomal aneuploidies, such as monosomy or trisomy. Individuals with Down syndrome, characterized by trisomy 21, have an approximately 40-fold increased risk of developing autism spectrum disorder [11]; therefore, an increased number of sex chromosomes is associated with a higher susceptibility to ASD. The X and Y chromosomes are implicated in the maturation of brain functions and in the development of neural networks involved in social, adaptive, cognitive, and behavioral processes [42]. Several studies have consistently shown that Sex Chromosome Trisomies (SCTs) (47,XXX, 47,XXY, 47,XYY) have a significant impact on the development of ASD symptoms. The prevalence of ASD is higher in SCTs, affecting 15% (range: 10.8–20%) of individuals with 47,XXX, 18% (range: 10–27%) of those with 47,XXY, and 30% (range: 19–43%) of those with 47,XYY [42]. Turner syndrome, although it is the only non-lethal monosomy, is not associated with an increased risk of ASD [11]. As further documented, standard chromosome analysis, fragile X DNA testing, and chromosomal microarrays were useful for diagnosing ASD in a cohort of 900 patients [13].

CNVs are genomic deletions or duplications of DNA segments that can affect gene dosage and contribute to disease susceptibility. Deleterious CNVs are found in as many as 20% of people with autism [43]. Conventional karyotyping and array-based comparative genomic hybridization (array-CGH) enable the detection of chromosomal abnormalities and CNVs, which account for approximately 5–10% of ASD cases [9]. They can be detected using chromosomal microarray analysis (CMA), which is considered the first-line test for children with NDDs [21]; using CMA, the diagnostic yields for ASD and other neurodevelopmental disorders in suspected patients are increased by approximately 20%, particularly in cases with suspected mosaicism [44].

CMA provides high-resolution genome-wide analysis and can identify submicroscopic chromosomal alterations below 100 kb, and it has revealed an elevated prevalence of CNVs in individuals with ASD. Prior studies have indicated that most apparently pathogenic CNVs in individuals with autism arise de novo [45]. Recent studies further emphasize that CNVs often act within a broader genomic context, where additional rare sequence variants or regulatory alterations can modulate penetrance and phenotypic variability, supporting the multiple-hit model in ASD pathogenesis [46,47].

#### Recurrent CNVs

As outlined in several studies, multiple chromosomal regions have been implicated in ASD. Among these, deletions within the 15q11–q13 region—associated with Angelman syndrome or Prader–Willi syndrome—as well as the 15q11.2 BP1–BP2 microdeletion linked to Burnside–Butler syndrome, represent well-established genomic loci contributing to ASD susceptibility [13]. The 22q11.2 deletion causes Velo-Cardio-Facial syndrome, and the 22q13 deletion is associated with Phelan–McDermid syndrome. These deletions fall into the category of “syndromic autism”, in which patients present not only with autism but also with congenital anomalies, facial dysmorphisms, and other clinical manifestations. These microdeletion-associated syndromes almost always include intellectual disability, early developmental delays, muscle hypotonia, and other clinically recognizable manifestations [45]. Other chromosomal regions reported with microdeletions or duplications include 1q24.2, 2q37.3, 3p26.2, 4q34.2, 6q24.3, 7q35, 13q13.2–q22, 15q11-q13, 15q22, 16p11.2, 17p11.2, 22q11, 2q13, and Xp22 [13].

Douard et al. analyzed how CNVs could influence intellectual quotient and autism risk [43]. They used the pLI (probability of being loss-of-function-intolerant) score, which measures the probability that a gene is intolerant to loss of function. Genes with a pLI probability of 80–90% were considered loss-of-function-intolerant. They found that an increase in the pLI of genes affected by CNVs, whether deletions or duplications, may increase the risk of autism. They also observed that pLI-measured haploinsufficiency elevates autism susceptibility across the genome, although much of this effect is mediated by NVIQ (Nonverbal Intelligence Quotient). The model was trained on deletions and duplications affecting over 4500 genes in both autism and unselected populations, and it was used to estimate that, on average, a 1 Mb coding deletion or duplication anywhere in the genome increases autism susceptibility, with median odds ratios of 1.6 and 1.3, respectively. This study reported that CNVs, measured using the pLI score, do not affect core autism symptoms. In contrast, CNVs strongly influence traits that contribute to the heterogeneity of the disorder, such as social communication (SRS scores), language, and motor abilities [43].

A high-impact study investigating the association between CNVs and ASD identified 36 genomic loci harboring ASD-associated genes [48]. The CNVs detected included both rare de novo and inherited events and frequently exhibited variable expressivity. Genes prioritized in this study included *CHD2*, *HDAC4*, and *GDI1*—previously implicated in other neurodevelopmental disorders—as well as additional candidate genes, such as *SETD5*, *MIR137*, and *HDAC9*. Another important finding of this study, consistent with the hypothesis of sex-specific modulators, was that females with ASD more frequently harbor highly penetrant CNVs (*p* = 0.017) and were overrepresented among individuals with fragile X syndrome protein targets (*p* = 0.02) [48]. Furthermore, genes disrupted by de novo CNVs and/or loss-of-function single-nucleotide variants were found to converge on functional networks involved in neuronal signaling and development, synaptic function, and chromatin regulation. Notably, one locus (11q13.2–q13.4) contained two ASD-associated genes (*SHANK2* and *KMT5B*), underscoring that genomic disorder loci may confer risk through the combined effects of multiple genes [48,49]. These findings illustrate that recurrent CNV regions are not monogenic in effect; rather, they often harbor multiple contributing genes whose additive or interactive effects influence both core ASD traits and broader neurodevelopmental outcomes [50,51]. These findings suggest that ASD-associated copy number variant regions can harbor more than one contributing gene, including genes with modest individual effect sizes that may be difficult to detect in isolation.

**Figure 2:**
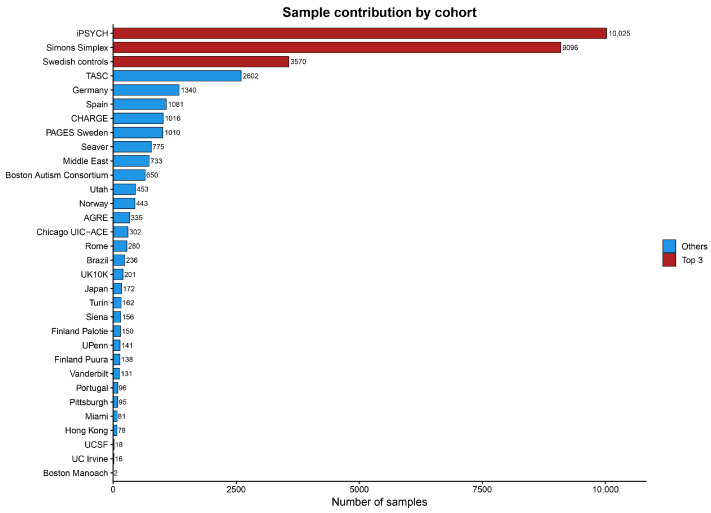
A bar plot showing the number of samples contributed by each cohort included in the largest WES study of 35,584 individuals, in which twelve genomic disorder loci encompassing a total of 13 genes associated with ASD were identified [49]. The cohorts are ordered by increasing sample size, with the largest contributors displayed at the top. The three largest cohorts (iPSYCH, Simons Simplex Collection, and Swedish controls) are highlighted in red, while all other cohorts are shown in blue. The total number of samples contributed by each cohort is indicated next to each bar.

As reported in the largest whole-exome sequencing study to date, involving 35,584 individuals, twelve genomic disorder loci were found to encompass a total of 13 genes associated with ASD. Figure 2 shows the number of samples contributed by each cohort included in this study.

CNVs affecting these loci were stratified into three groups based on how ASD risk genes were distributed within each genomic region [49]. In the first group, the ASD-associated gene represented the primary gene implicated within the locus, as illustrated by *SHANK3* in Phelan–McDermid syndrome. In the second group, the ASD risk gene differed from the gene initially considered most relevant within the region, exemplified by HDLBP at the 2q37.3 locus. In the third group, ASD-associated genes were identified within loci where no single gene had previously been clearly implicated, such as *BCL11A* at 2p15–p16.1.

It is noteworthy that CNVs are frequently accompanied by additional genetic alterations, and this condition, described as the multiple-hit model, can substantially influence phenotypic variability and clinical severity. The presence of secondary variants, including SNVs and other rare sequence variants affecting functionally relevant genes, contributes additively to ASD and cognitive disability risk. An increased burden of such secondary hits correlates with more severe clinical manifestations. Therefore, the pathogenic effect of a CNV is not determined by the CNV alone but rather by the global genomic context in which it occurs. Moreover, environmental factors may further modulate the severity of CNV-associated phenotypes, contributing to the onset and variability of clinical manifestations [45]. Table 2 provides an overview of representative genomic loci, including deletions and duplications, their genomic sizes, associated clinical features, and supporting references.

### 3.2. Genes Associated with ASD

Several authors emphasize that the genetic architecture of ASD cannot be explained by either rare or common variants alone [72]. Instead, ASD risk arises from the combined contribution of rare, high-impact variants and numerous common variants with individually small effect sizes. Although rare variants are observed at low population frequencies, they often confer substantial individual risk, whereas common variants collectively account for a significant proportion of genetic liability. In addition, environmental and epigenetic modifiers likely interact with this genetic background. Consistent with the aim of this review, the current evidence supports a multifactorial model in which multiple genetic and non-genetic factors jointly contribute to ASD susceptibility. Based on these considerations, although environmental factors and epigenetic modifications play a pivotal role in ASD etiology, a clear understanding of the genes involved remains essential. Although there is broad consensus that ASD represents a complex clinical phenotype with a multifactorial etiology, several monogenic disorders with autosomal or X-linked inheritance may include ASD among their clinical manifestations [13,73]. Examples include tuberous sclerosis complex (*TSC1*, *TSC2*), neurofibromatosis (*NF1*, *NF2*), Rett syndrome (*MECP2*), and fragile X syndrome (*FMR1*) (Table 3).

Additional syndromic conditions associated with ASD features include Sotos, Noonan, Möbius, Cohen, Cornelia de Lange, and Joubert syndromes, myotonic dystrophy, and the oculo-auriculo-vertebral spectrum, as well as *PTEN*-related disorders characterized by extreme macrocephaly. Although these conditions are not defined as autism per se, they share overlapping neurodevelopmental and behavioral features with ASD.

In the largest ASD exome sequencing study to date, involving 35,584 individuals (including 11,986 with ASD), 102 ASD risk genes were identified at a false discovery rate (FDR) ≤ 0.1 [49]. Among these genes, 49 were enriched for disruptive de novo variants in individuals with severe neurodevelopmental delay, whereas 53 were more frequently disrupted in ASD-ascertained individuals, highlighting phenotype-specific genetic contributions. Notably, the majority of ASD risk genes converge on a limited number of biological pathways, primarily involving the regulation of gene expression and neuronal communication. A large subset of these genes encodes chromatin remodelers (GO:0006338) and transcriptional regulators (GO:0003713; GO:0006357) (e.g., *ADNP*, *ARID1B*, *ASH1L*, *CHD2*, *CHD8*, *KMT5B*, *MED13L*, *POGZ*, and *SUV420H1*), which play critical roles in controlling gene expression programs during early brain development. Another major group includes genes involved in synaptic function and neuronal signaling, such as *GRIN2B*, *SHANK3*, *SYNGAP1*, and *SCN2A*, which are essential for synaptic transmission (GO:0050803), plasticity (GO:0048167), and network excitability. Additionally, genes like *FOXP1* and *RBFOX1* regulate RNA processing and alternative splicing (GO:0008380), further contributing to neuronal differentiation and function (GO:0007399). Other ASD-associated genes, including *PTEN* and *DYRK1A*, are involved in intracellular signaling pathways that control cell growth, proliferation, and neuronal maturation. Collectively, these findings highlight that ASD risk genes predominantly affect pathways governing transcriptional regulation, synaptic organization, and neuronal signaling rather than represent functionally unrelated entities [49].

In a large-scale whole-genome sequencing study of 2308 individuals from multiplex ASD families, 69 genes associated with ASD risk were identified, including 24 genes reaching genome-wide significance after Bonferroni correction and 16 newly implicated risk genes, predominantly supported by rare inherited variants [74]. Enrichment analyses revealed that these inherited risk genes were mainly involved in cytoskeletal organization and ion transport pathways, which differ from the pathways typically implicated by de novo variant-driven ASD studies. Despite these differences, both inherited and de novo ASD risk genes converged on a shared protein–protein interaction network, suggesting that distinct classes of genetic variation ultimately perturb common molecular and cellular processes. Beyond coding variation, the study also identified structural variants affecting non-coding regulatory regions, including recurrent deletions in the promoter regions of *DLG2* and *NR3C2*. Functional validation in zebrafish demonstrated that loss of nr3c2 disrupted sleep and social behaviors, phenotypes overlapping with core ASD-related traits in humans [75].

In another pivotal study investigating the developmental consequences of haploinsufficiency in three ASD risk genes—*KMT5B*, *ARID1B*, and *CHD8*—the authors employed human cerebral cortex organoid models [76]. The study demonstrated that the haploinsufficiency of each gene induced the asynchronous development of two major cortical neuronal lineages, namely, γ-aminobutyric acid (GABA)-ergic interneurons and deep-layer excitatory projection neurons. Notably, although these genes converged on similar neurodevelopmental phenotypes, they acted through largely distinct molecular pathways. Nevertheless, all of these genes were associated with abnormal circuit activity during the early stages of brain development. Another study systematically investigated whether individuals with ASD carrying variants in 13 well-established ASD risk genes also exhibited brain MRI abnormalities [77]. Notably, all individuals harboring *ARID1B* variants showed detectable brain MRI abnormalities.

A recent study using the CRISPR–human organoids–single-cell RNA sequencing (CHOOSE) platform examined 36 high-risk ASD genes involved in transcriptional regulation and showed that their perturbation disrupts cell fate determination [78]. Dorsal and ventral progenitors, as well as upper-layer excitatory neurons, were identified as the most vulnerable cell types. Notably, mutation of the BAF complex subunit *ARID1B* altered progenitor differentiation toward oligodendrocyte and interneuron precursor lineages, a finding validated in patient-derived induced pluripotent stem cell organoids. Overall, this approach highlights the value of high-throughput organoid models for linking ASD risk genes to cell-type-specific developmental trajectories [78].

Several curated databases compile extensive lists of genes associated with ASD. Among these, the SFARI Gene database represents one of the most comprehensive and up-to-date resources. As noted in Section 1, this dataset provides a structured, evidence-based gene scoring system to classify ASD-associated genes. According to its most recent update (January 2026), the database includes more than 1200 candidate genes annotated and ranked based on the strength of supporting evidence.

According to the MalaCards database, a total of 1758 genes have been associated with ASD to date. Notably, the top-ten tier of inferred genes—*CHD8*, *GRIN2B*, *TSC2*, *NRXN1*, *MEF2C*, *CHD2*, *POGZ*, *TRAPPC9*, *DCC*, and *KAT6A*—exhibits association scores exceeding 500, highlighting their strong relevance to ASD pathogenesis. Among these genes, *NRXN1* encodes a presynaptic cell adhesion molecule essential for synapse formation and maintenance, thereby regulating excitatory neurotransmission and synaptic connectivity [79]. *TRAPPC9* is involved in intracellular trafficking and NF-κB signaling, contributing to neuronal differentiation and brain development [80], while *KAT6A* functions as a histone acetyltransferase, playing a key role in chromatin remodeling and the epigenetic regulation of gene expression during neurodevelopment [81]. Finally, *DCC* encodes a receptor for netrin-1 that is crucial for axon guidance and the establishment of long-range neuronal connections [82]. According to the DOMINO inheritance prediction tool, among these genes, *TRAPPC9* is the only one predicted to have a very likely recessive mode of inheritance, whereas all the remaining genes are predicted to follow a very likely dominant inheritance pattern. This observation is consistent with previous studies indicating that ASDs are predominantly caused by de novo mutations or by dominant inheritance from asymptomatic carriers of such variants [83,84]. This observation can be explained by the high intolerance to loss-of-function (LoF) variants exhibited by several ASD-associated genes, as reflected by the elevated probability of loss-of-function intolerance (pLI) scores. Recent NGS studies highlight that rare de novo and inherited variants in high-pLI genes, such as *CHD8*, *GRIN2B*, and *ARID1B*, converge on shared molecular pathways regulating synaptic development, chromatin remodeling, and neuronal signaling, reinforcing the central role of these genes in ASD etiology [46,47,85]. For example, according to the OMIM database, *CHD8* is associated with intellectual developmental disorder with autism and macrocephaly (MIM #615032), inherited in an autosomal dominant manner. Constraint metrics from both the Genome Aggregation Database (gnomAD) and DECIPHER database further show that *CHD8* has a very high pLI score and a markedly low loss-of-function observed/expected upper-bound fraction (LOEUF), indicating strong selective constraint against LoF variants in the general population, suggesting that such variants are poorly tolerated and likely to have severe functional consequences. Consequently, pathogenic LoF variants in *CHD8* are more likely to arise as de novo events or be inherited from asymptomatic carriers with incomplete penetrance, thereby contributing disproportionately to ASD risk despite their rarity. This aspect is supported by several studies that have investigated *CHD8* haploinsufficiency in individuals with ASD [86,87]. These studies demonstrate that a reduced *CHD8* dosage disrupts early brain development, affecting processes such as neuronal proliferation, differentiation, and cortical patterning while also impairing protein homeostasis and cellular regulatory mechanisms later in life [88]. A similar pattern is observed for *GRIN2B*, a gene associated in the OMIM database with both developmental and epileptic encephalopathy (MIM #616139) and intellectual developmental disorder with or without seizures (MIM #613970) with autosomal dominant inheritance. As indicated by both the DECIPHER and gnomAD databases, *GRIN2B* also displays a high pLI score and low LOEUF value, indicating strong intolerance to loss-of-function variants. This suggests that disruptive variants in *GRIN2B* are likely to have substantial functional consequences and contribute to ASD risk, often through de novo events. This aspect has been corroborated by various studies [89].

Several authors have emphasized the presence of a shared molecular etiology between ASD and other neurodevelopmental and psychiatric disorders, including schizophrenia (SCZ), bipolar disorder (BD), and obsessive–compulsive disorder (OCD) [90,91]. Converging evidence suggests that this overlap is largely driven by alterations in neurotransmission, synaptic plasticity, and intracellular signaling pathways. In particular, genes such as *DRD2* and *SLC6A3* are involved in dopaminergic signaling (GO:0014059), whereas *HTR2A* and *TPH2*, together with the monoamine oxidase gene *MAOA*, regulate serotonergic neurotransmission (GO:0099589) and monoamine metabolism (GO:0097621). Additionally, *CACNA1C* and *CHRNA7* contribute to neuronal excitability and synaptic transmission through voltage-gated calcium channels (GO:0008331) and cholinergic receptor activity (GO:0015464), respectively. Axon guidance (GO:0051378; GO:0007411) and synaptic assembly (GO:0007416) and plasticity are further influenced by *BDNF*, while MTHFR plays a role in folate metabolism (GO:0051593) and methylation processes, thereby impacting epigenetic regulation. Finally, *NOS1AP* has been implicated in nitric oxide signaling (GO:0030235) and glutamatergic synaptic activity (GO:0098978). Together, these mechanisms highlight how disruptions in neurotransmitter systems, intracellular signaling, and gene regulation pathways may underlie the shared genetic architecture across ASD, SCZ, BD, and OCD. These findings highlight the intricate interplay between neural development, synaptic processing, and molecular regulation in the etiology of ASD.

### 3.3. Epigenetic Modifications

Genetic heritability alone does not fully account for the manifestation of ASD; epigenetic regulation represents an additional critical layer influencing gene expression without altering the underlying DNA sequence. Currently, a definitive genetic etiology is identified in approximately 25–35% of individuals with ASD, reflecting the intrinsic limitations of the existing diagnostic technologies [92]. Epigenetic mechanisms include a wide range of chemical modifications affecting DNA and chromatin structure, such as histone acetylation, methylation, phosphorylation, and ubiquitination, as well as DNA methylation at CpG islands. These modifications modulate chromatin accessibility and transcriptional activity: histone modifications can either activate or repress gene expression depending on the specific residue and genomic context, whereas DNA methylation at promoter-associated CpG islands is generally associated with transcriptional repression [93,94]. Altered histone acetylation or DNA methylation at CpG islands can disrupt neurodevelopment, potentially leading to autistic traits like social, sensory, and language difficulties.

Several maternal conditions may affect the onset of autism, including pre-pregnancy BMI, diabetes, and hypertension [95,96]. In addition to genetic and epigenetic factors, prenatal environmental exposures have been increasingly implicated in ASD risk. Perinatal exposure to environmental pollutants, pesticides, and other chemical agents has been associated with neurodevelopmental vulnerability, potentially through mechanisms involving DNA damage, oxidative stress, and epigenetic dysregulation [97]. Advanced maternal age may further amplify susceptibility by increasing the accumulation of environmental toxins and germline mutations, as well as promote DNA hypermethylation patterns linked to altered gene expression. Nutritional factors also play a critical role: micronutrients such as zinc, copper, iron, and vitamin B9 (folate) have been proposed as modulators of ASD etiology due to their involvement in neurodevelopment, methylation pathways, and synaptic function [98]. Moreover, exposure to specific toxicants may trigger maternal immune activation and inflammatory responses, leading to fetal micronutrient imbalances that disrupt early brain development and neuronal maturation.

Pre-pregnancy obesity is associated with an increased risk of having children with ASD [99]. Obesity induces accumulation of macrophages and lipids in the placenta during pregnancy, causing placental inflammation and oxidative stress, affecting fetal brain development and reprogramming. These placental morphological alterations can disrupt epigenetic processes, such as DNA methylation and hydroxymethylation, histone acetylation, and microRNA regulation, ultimately resulting in altered fetal brain programming during gestation. Within this context, mercury exposure has been proposed as a potential factor linking maternal obesity to an increased risk of ASD [100].

Among the maternal risk factors implicated in ASD, advanced maternal age has been associated with a higher incidence of obstetric complications, such as preterm birth, low birth weight, labor complications, cesarean delivery, and chromosomal anomalies [101]. Mothers with type 1, type 2, or gestational diabetes have a 62% higher risk of having children with ASD [99]. Given the association between ASD and diabetes, future research should focus on personalized, neurodiversity-informed approaches to diabetes self-management in autistic individuals [102]. Co-produced, patient-centered models integrating physical and mental health will be essential to improve long-term outcomes.

Prenatal stress can interact with genetic factors, increasing the risk of ASD in offspring. Several studies have highlighted that mutations in *SLC6A4*, when combined with maternal stress during pregnancy, may play a significant role in the development of ASD. Moreover, studies on animal models and intergenerational data suggest that maternal stress can modulate gene expression and influence behavioral traits in offspring. In this context, the combination of genetic predisposition and prenatal stress exposure represents an important risk factor for the development and severity of ASD [96].

Studies have shown that maternal dysfunctions can alter the levels of *DNMT3a* and *DNMT3b*. Changes in these enzymes can lead to inadequate DNA methylation and imprinting defects in the embryo and offspring, potentially contributing to the development of neurodevelopmental disorders, such as ASD [96]. These observations support a model in which genetic susceptibility interacts with epigenetic and environmental factors—such as maternal obesity, prenatal stress, or micronutrient imbalances—to influence neuronal differentiation, synaptic connectivity, and the emergence of ASD-related behavioral phenotypes [103,104].

## 4. Signaling Pathways

The scientific community widely recognizes that ASD results from a complex interaction among genetic, environmental, and immunological factors [72,105]. The identification of ASD-related signaling pathways may substantially advance understanding of ASD pathophysiology and facilitate the discovery of precise molecular targets for autism. Increasing evidence indicates that the major cellular pathways implicated in ASD are tightly interconnected and regulated by neuronal activity, particularly during brain development. Among these, as outlined in a prior relevant review, there is an established convergence between the core pathway synaptic function, WNT signaling, and translational control, which have emerged as central to neurodevelopment and ASD pathogenesis [106]. Synaptic signaling is a fundamental mechanism underlying learning and memory, as it enables the formation and refinement of neural networks through synaptic plasticity, particularly at excitatory synapses [107]. Excitatory synaptic transmission is mediated primarily by glutamate, which activates postsynaptic receptors to regulate neuronal connectivity and circuit strength. Accumulating evidence indicates that disruptions in this pathway contribute to ASD, including defects in RNA splicing that affect the expression and regulation of synaptic genes [108]. These splicing abnormalities can, in turn, interfere with the reciprocal regulation between translational control pathways and synaptic proteins, leading to altered synthesis, localization, and function of synaptic receptors and scaffolding proteins [109,110]. Together, these defects compromise synaptic development and plasticity, providing a mechanistic link between dysregulated gene expression and synaptic dysfunction in ASD. Additionally, several authors have described that defects in the dopaminergic pathway may lead to ASD susceptibility [111]. Other authors have highlighted the mTOR signaling pathway as another key pathway associated with ASD, given its pivotal role in brain development [112,113,114]. The following subsections provide further insight into the mechanistic links between the previously described signaling pathways and ASD pathogenesis. In particular, we focus on how genetic mutations affecting genes within these pathways can lead to a broad spectrum of neurodevelopmental phenotypes, including ASD. Additionally, we emphasize that these pathways converge at the level of shared genes, as several ASD-associated genes participate in multiple signaling cascades, linking synaptic function, WNT signaling, and mTOR-mediated translational control. Table S1 lists the main genes involved in the major ASD-related signaling pathways, indicating their SFARI gene scores, as well as the associated phenotypes and inheritance patterns annotated in the OMIM database.

### 4.1. WNT Signaling

The WNT signaling pathway (KEGG entry: map04310) is a highly conserved molecular cascade that plays a fundamental role in embryonic development and neurodevelopment, including neural progenitor proliferation, neuronal differentiation, migration, cortical patterning, dendritic growth, synapse formation, and activity-dependent plasticity. The pathway is classically divided into the canonical (β-catenin-dependent) and non-canonical (β-catenin-independent) branches, both of which are essential for proper brain development and have been strongly implicated in ASD [115]. WNT signaling regulates transcriptional programs that control neuronal maturation, growth, and neural circuit assembly during tightly regulated developmental windows, processes that are highly sensitive to dosage and timing. Disruption of this pathway can therefore result in widespread and pleiotropic neurodevelopmental consequences, consistent with the clinical and genetic heterogeneity observed in ASD [115]. Accordingly, dysregulated WNT signaling is thought to represent a key contributor to ASD etiology.

**Figure 3:**
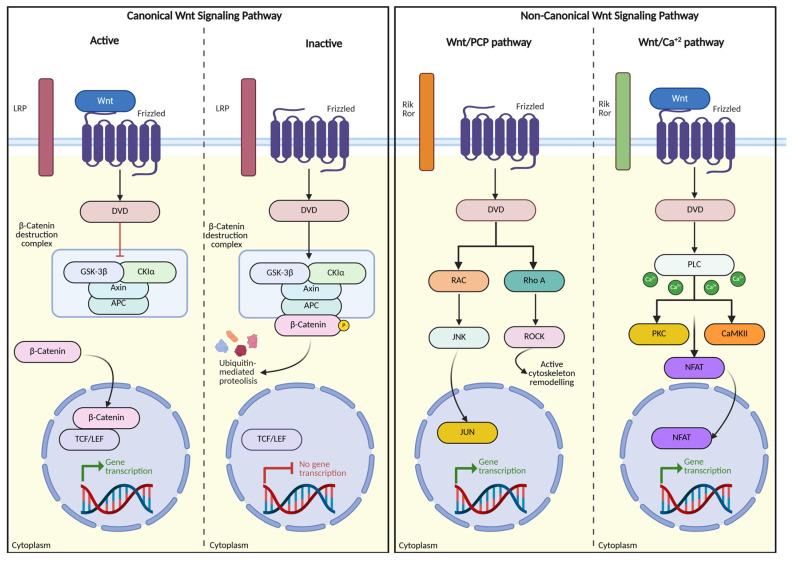
The canonical and non-canonical WNT signaling pathways. The figure illustrates the major branches of WNT signaling. Left panel: In the canonical WNT/β-catenin pathway, binding of WNT ligands to Frizzled and LRP5/6 receptors inhibits the β-catenin destruction complex (AXIN, APC, GSK3β, CK1α), allowing β-catenin stabilization and nuclear translocation, where it activates TCF/LEF-dependent gene transcription. In the absence of WNT, β-catenin is phosphorylated and targeted for ubiquitin-mediated degradation, preventing transcriptional activation. Right panel: non-canonical WNT signaling includes the WNT/PCP pathway, which activates RhoA/Rac–ROCK/JNK signaling to regulate cytoskeletal dynamics and cell polarity, and the WNT/Ca^2+^ pathway, which induces intracellular calcium release and activates PKC-, CaMKII-, and NFAT-dependent transcription. Together, these pathways regulate neuronal migration, synapse formation, and activity-dependent gene expression. Dysregulation of both canonical and non-canonical WNT signaling has been implicated in neurodevelopmental disorders, including autism spectrum disorder.

Genetic studies have identified multiple ASD-associated genes that directly or indirectly modulate WNT signaling. Among these, *CHD8* (SFARI score 1S) is a high-confidence syndromic ASD risk gene encoding a chromatin remodeler that regulates the transcription of WNT pathway components. Haploinsufficiency of *CHD8* leads to altered WNT target gene expression, abnormal brain overgrowth, disrupted cortical development, and recurrent phenotypes with dysmorphic facial features, supporting its classification as a syndromic form of ASD [116]. Similarly, *PTEN* (SFARI score 1S), another well-established ASD risk gene, negatively regulates the PI3K–AKT pathway, which functionally intersects with WNT signaling to control neuronal growth, synaptic stability, and circuit maturation. Pathogenic *PTEN* variants are frequently associated with macrocephaly and ASD. Additional ASD-associated genes, including *CTNNB1* (β-catenin), *APC*, *DVL*, and TCF/LEF transcriptional regulators, further support the involvement of canonical WNT/β-catenin signaling in ASD susceptibility [117,118]. Figure 3 illustrates both the canonical and non-canonical WNT signaling pathways.

### 4.2. Dopaminergic Pathway

Another pivotal system implicated in ASD pathogenesis is the central dopaminergic system. Accumulating evidence indicates that genetic variants affecting components of this pathway contribute to ASD susceptibility [111]. These genes encode dopamine receptors, transporters, and enzymes involved—either directly or indirectly—in dopamine synthesis, signaling, and regulation. The dopaminergic system, in combination with the serotoninergic system, is involved in neurotransmission, brain maturation and cortical organization, while neurotrophic factors (NTFs) participate in neurodevelopment, neuronal survival and synapsis formation [119].

For instance, *DRD1*, *DRD2*, and *DRD3* encode for dopamine receptor subtypes that are differentially expressed across the cortical and subcortical brain regions and play complementary roles in regulating neuronal excitability, synaptic plasticity, reward processing, and executive function [120,121]. Collectively, these genes have been associated with ASD. In fact, as reported in the SFARI Gene database, all three receptors are classified as strong ASD candidates (score 2), supporting convergent evidence for their involvement in ASD despite the absence of well-defined monogenic phenotypes for *DRD1* and *DRD2* in the OMIM database. Functionally, DRD1 and DRD2 represent the principal modulators of dopamine-dependent excitatory and inhibitory signaling, respectively, and are critically involved in synaptic plasticity, learning, cognitive control, and motor regulation [120]. Subtle dysregulation of these receptors—arising from common or rare genetic variants—may therefore contribute to ASD-related alterations in attention, executive functioning, reward sensitivity, and behavioral flexibility [121]. In contrast, *DRD3*, which exhibits a more restricted expression pattern and higher affinity for dopamine, has been associated with schizophrenia susceptibility (MIM #181500) and has been repeatedly implicated in neurodevelopmental disorders, including ASD and ADHD, particularly in relation to repetitive and stereotyped behaviors [122,123,124]. Importantly, the involvement of DRD1–3 in ASD supports a model in which dopaminergic dysfunction contributes to ASD risk through the quantitative modulation of neural circuits rather than through highly penetrant, single-gene disorders. This interpretation aligns with the broader polygenic and pathway-based genetic architecture of ASD, in which multiple genes affecting neurotransmitter systems converge on shared neural processes underlying social behavior, cognition, and behavioral regulation. Together, these findings reinforce the dopaminergic system as a biologically coherent and clinically relevant pathway in ASD pathophysiology.

The DDC gene encodes aromatic L-amino acid decarboxylase, a key enzyme in monoamine neurotransmitter biosynthesis that catalyzes the conversion of L-DOPA to dopamine and 5-hydroxytryptophan to serotonin. According to the Online Mendelian Inheritance in Man (OMIM), biallelic pathogenic variants in DDC cause aromatic L-amino acid decarboxylase deficiency (MIM #608643), a rare neurodevelopmental disorder inherited in an autosomal recessive manner. In addition to its role in monogenic disease, DDC has been implicated in ASD, with a significant association observed in single-marker analyses [119]. Consistent with this evidence, DDC is classified as a strong ASD candidate (score 2) in the SFARI Gene database.

Some members of the solute carrier (SLC) family play a central role in dopaminergic neurotransmission by regulating the transport of dopamine and related monoamines across cellular membranes. In particular, dopamine transporters encoded by SLC genes control synaptic dopamine availability through reuptake mechanisms, thereby shaping signal duration and intensity. Genetic variation in several SLC family members has been implicated in ASD and other neurodevelopmental conditions, including attention-deficit/hyperactivity disorder, intellectual disability, and movement disorders [78,125]. Dysregulation of SLC-mediated transport during critical periods of brain development may alter dopaminergic signaling, affecting neural circuit maturation, reward processing, and behavioral regulation. Owing to their central role in neurotransmission, several SLC genes have therefore been proposed as potential therapeutic targets. Although numerous SLC genes have been associated with ASD [126], not all are directly involved in dopamine transport. Among dopamine-related SLC genes, *SLC6A3* and *SLC29A4* are currently annotated as strong ASD candidates (SFARI score = 2) in the SFARI Gene database. The *SLC6A3* gene encodes the dopamine transporter (DAT), a sodium-dependent amine transporter responsible for terminating dopaminergic signaling through the high-affinity reuptake of dopamine into presynaptic terminals [127]. According to the OMIM database, biallelic pathogenic variants in *SLC6A3* cause infantile parkinsonism-dystonia (MIM #613135; autosomal recessive), and variants have also been associated with susceptibility to nicotine dependence (MIM #188890). Beyond monogenic disorders, SLC6A3 has emerged as a multidisease risk gene implicated in a broad spectrum of neuropsychiatric and neurological conditions. Both rare and common variants affecting DAT function have been linked to alcohol use disorder (high-activity variants), ADHD (low-activity variants), ASD—potentially through disrupted protein interaction networks—and movement disorders involving regulatory or familial mutations [121,127,128].

### 4.3. mTOR

Among intracellular signaling cascades, the mTOR signaling pathway plays a pivotal role not only in cell-cycle regulation but also in protein synthesis and the maintenance of brain homeostasis, and it has been strongly implicated in ASD [129]. mTOR signaling is essential during brain development, where it regulates neural progenitor proliferation, neuronal growth, dendritic arborization, synapse formation, and synaptic plasticity. Dysregulation of this pathway—resulting from either hyperactivation or impaired inhibitory control—has been observed in both syndromic and non-syndromic forms of ASD. Notably, pathogenic variants in key mTOR regulators, such as *PTEN*, *TSC1*, and *TSC2*, lead to aberrant mTOR activation and are frequently associated with ASD, often in conjunction with macrocephaly, epilepsy, and intellectual disability. In addition to these well-defined genetic conditions, altered mTOR signaling has also been proposed as a contributing mechanism in idiopathic ASD, supporting a broader role for mTOR pathway dysfunction in ASD pathophysiology [129]. As previously discussed in Section 4.1, the *PTEN* gene is also involved in WNT signaling, and germline mutations affecting *PTEN* have been consistently implicated in ASD and intellectual disability, defining a distinct molecular and clinical subtype of the condition [115,130]. PTEN is pivotal for its functions as a major negative regulator of the phosphatidylinositol 3-kinase (PI3K)/AKT/mTOR signaling pathway, thereby controlling key cellular processes such as growth, protein synthesis, proliferation, and survival [131]. Loss-of-function mutations in PTEN result in hyperactivation of the PI3K/AKT/mTOR cascade, leading to abnormal neuronal growth, altered synaptic development, and disrupted neural circuit formation. PTEN-associated ASD is characterized by a distinctive clinical phenotype, most notably macrocephaly or extreme brain overgrowth, frequently accompanied by intellectual disability, seizures, and behavioral features consistent with ASD. These features highlight the critical role of tightly regulated mTOR signaling in normal neurodevelopment. As emphasized by multiple studies, PTEN-associated ASD provides a paradigmatic example of how dysregulated intracellular signaling can drive ASD pathophysiology and underscores the relevance of pathway-based mechanisms linking syndromic and non-syndromic forms of ASD [131,132]. Evidence from animal models demonstrates that dysregulation of the PI3K/Akt/mTOR signaling pathway can give rise to neuropsychiatric phenotypes that closely resemble core features of ASD, including impairments in social interaction, repetitive behaviors, and altered cognitive function [8,133]. These findings suggest that abnormal PI3K/Akt/mTOR signaling disrupts activity-dependent synaptic development and neural circuit maturation, providing mechanistic insight into how pathway imbalance contributes to ASD-related behavioral abnormalities.

### 4.4. Glutamatergic and GABAergic Pathways

Glutamatergic neurotransmission and GABAergic neurotransmission represent two molecularly distinct but functionally interdependent signaling pathways that are essential for normal brain development and function [134]. The glutamatergic system provides the principal excitatory drive in the central nervous system, supporting neuronal activation, synaptic plasticity, learning, and memory, whereas the GABAergic system exerts inhibitory control over neuronal excitability, stabilizing neural circuits and maintaining network homeostasis. The precise balance between excitation and inhibition (E/I balance) emerging from the coordinated activity of these two systems is critical for proper neurodevelopment, and disruption of this balance is a well-established neurobiological hallmark of ASD [135,136].

**Figure 4:**
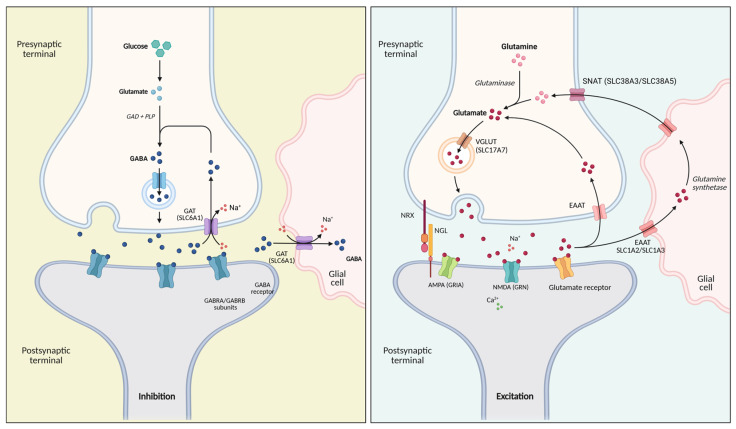
A schematic representation of inhibitory GABAergic and excitatory glutamatergic synaptic transmission and associated molecular components. The figure illustrates the main molecular pathways underlying inhibitory and excitatory neurotransmission and highlights mechanisms relevant to neurodevelopmental disorders and ASD. The left panel shows the inhibition mechanism involving GABAergic synaptic transmission, depicting the conversion of glutamate into γ-aminobutyric acid (GABA) through glutamate decarboxylase (GAD) and pyridoxal phosphate (PLP). GABA is released from the presynaptic terminal and binds to postsynaptic GABA receptors composed of GABRA/GABRB subunits, mediating inhibitory signaling. GABA reuptake is regulated by sodium-dependent transporters (GAT; SLC6A1) located on neuronal and glial membranes, contributing to neurotransmitter clearance and synaptic homeostasis. In contrast, the right panel shows the excitation mechanism involving the glutamatergic synaptic transmission. This mechanism includes the glutamine uptake via SNAT transporters (SLC38A3/SLC38A5), the conversion to glutamate by glutaminase, and vesicular loading through VGLUT (SLC17A7). Released glutamate activates ionotropic AMPA (GRIA) and NMDA (GRIN) receptors as well as metabotropic glutamate receptors on the postsynaptic membrane, promoting excitatory signaling and calcium influx. Excitatory amino acid transporters (EAAT; SLC1A2/SLC1A3) regulate glutamate reuptake in neurons and glial cells, maintaining synaptic balance.

Despite their distinct molecular architectures, the glutamatergic and GABAergic pathways are tightly interconnected at both the metabolic and synaptic levels [137]. Glutamate serves not only as the primary excitatory neurotransmitter but also as the direct biochemical precursor of γ-aminobutyric acid (GABA), which is synthesized through the action of glutamate decarboxylase enzymes encoded by several enzymes, such as GAD1 and GAD2. Both *GAD1* and *GAD2* have been reported as susceptibility genes for several psychiatric disorders, including schizophrenia, anxiety, and panic-related conditions [138,139]. Notably, epigenetic regulation—particularly DNA methylation changes affecting these genes—has been proposed as a potential molecular signature associated with altered GABAergic function [140,141]. This metabolic coupling is further reinforced by astrocyte-mediated neurotransmitter recycling, whereby glutamate released at excitatory synapses is taken up by excitatory amino acid transporters (SLC1A2 and SLC1A3), converted into glutamine, and shuttled back to neurons via system N/A transporters of the SLC38 family. Glutamine is subsequently reconverted to glutamate and, in inhibitory neurons, to GABA, establishing a shared neuron–glia metabolic network linking excitatory and inhibitory neurotransmission. Figure 4 schematizes both inhibitory GABAergic and excitatory glutamatergic synaptic transmission.

At the synaptic level, the two pathways rely on distinct vesicular transporters and receptor systems. Glutamatergic neurons package glutamate into synaptic vesicles via vesicular glutamate transporters (SLC17A6/7/8), activating ionotropic and metabotropic glutamate receptors on postsynaptic neurons. In contrast, GABAergic neurons load GABA into vesicles via the vesicular GABA transporter (SLC32A1) and signal primarily through GABAA and GABAB receptors to suppress neuronal firing. The clearance mechanisms also differ, with glutamate removed mainly by EAAT transporters and GABA by GAT transporters such as SLC6A1 [142,143]. Disruption of these tightly regulated processes may alter neurotransmitter availability and synaptic strength, thereby destabilizing E/I balance.

During early brain development, GABA initially exerts depolarizing, excitatory effects before transitioning to inhibitory signaling, a developmental switch that is essential for neuronal maturation, synapse formation, and circuit refinement [135]. Perturbations affecting GABA synthesis, receptor composition, synaptic transmission, or chloride homeostasis can interfere with this trajectory, resulting in abnormal network activity. In parallel, defects in glutamatergic signaling have been shown to impact the RNA processing and translational regulation of synaptic proteins, further contributing to synaptic dysfunction in ASD [108]. These alterations highlight the reciprocal relationship between synaptic signaling and gene expression control in neurodevelopmental disorders.

Genetic studies have identified ASD-associated variants affecting both the glutamatergic and GABAergic systems, as well as genes operating at their interface. Variants in glutamatergic genes, such as *GRIN2B*, *GRIA*, and *SLC17A7*, and in GABAergic genes, including *GABRA1*, *GABRB3*, *GAD1/2*, and *SLC6A1*, have been repeatedly implicated in ASD and related neurodevelopmental phenotypes. For instance, genetic variants in *GRIN2B* have been associated with a broad spectrum of neurodevelopmental disorders, including ASD [89,144]. According to the OMIM database, *GRIN2B* is linked to developmental and epileptic encephalopathy (MIM #616139) and intellectual developmental disorder with or without seizures (MIM #613970), both inherited in an autosomal dominant manner. Similarly, *SLC6A1* has been implicated in several psychiatric and neurodevelopmental conditions. OMIM annotations include associations with anxiety-related personality traits (MIM #607834) and obsessive–compulsive disorder (MIM #164230), also with autosomal dominant transmission. In addition, pathogenic variants in *SLC6A1* have been increasingly reported in neurodevelopmental disorders encompassing ASD [145,146]. Moreover, synaptic adhesion and scaffolding genes such as *NRXN1* and *CNTNAP2* influence the formation and maintenance of both excitatory and inhibitory synapses, underscoring the convergence of these pathways at the level of neural circuit assembly. As widely reported, pathogenic variants in *NRXN1* have been associated with ASD as well as with other psychiatric conditions, including schizophrenia [147,148]. According to the OMIM, NRXN1 is annotated as a schizophrenia susceptibility locus (MIM #621407) and as the causative gene for Pitt–Hopkins-like syndrome 2 (MIM #614325), an autosomal recessive neurodevelopmental disorder often presenting with autistic features.

Overall, these findings support a model in which the glutamatergic and GABAergic pathways, while molecularly distinct, converge on shared developmental and functional processes regulating neuronal network stability. Disruption of either pathway—or of their coordinated interaction—can shift excitation–inhibition balance toward hyperexcitability or excessive inhibition, leading to altered synaptic plasticity, abnormal connectivity, and behavioral phenotypes characteristic of ASD. This convergence places E/I imbalance at the core of ASD pathophysiology and provides a unifying framework linking genetic susceptibility, synaptic dysfunction, and neurodevelopmental outcomes.

### 4.5. MAPK Signaling Pathway

The mitogen-activated protein kinase (MAPK) signaling pathway represents a key intracellular cascade regulating neuronal proliferation, differentiation, synaptic plasticity, and activity-dependent gene expression during brain development [149]. Increasing evidence suggests that dysregulation of MAPK signaling contributes to the molecular mechanisms underlying ASD, particularly through its interaction with synaptic and translational control pathways. The MAPK/ERK branch, activated downstream of receptor tyrosine kinases and synaptic glutamatergic signaling, plays a central role in translating extracellular stimuli into transcriptional programs that shape neuronal maturation and circuit formation [150].

Genetic studies have highlighted several ASD-associated genes that either directly regulate or function downstream of MAPK signaling. Variants affecting components of the RAS–MAPK cascade are frequently observed in neurodevelopmental conditions collectively referred to as RASopathies, including neurofibromatosis type 1 (NF1), Noonan syndrome (PTPN11, SOS1), and cardio-facio-cutaneous syndrome (RAF1, KRAS), which often present with autistic traits and cognitive impairment [151]. In addition, synaptic genes strongly associated with ASD, such as *SYNGAP1* and *SHANK3*, modulate ERK activation at excitatory synapses, linking glutamatergic neurotransmission with intracellular signaling pathways that control synaptic strength and plasticity [152].

Pathogenic variants in *SYNGAP1* have been extensively documented in the literature and are strongly associated with neurodevelopmental disorders, including ASD and intellectual disability [153,154]. According to the OMIM, *SYNGAP1* is linked to intellectual developmental disorder (MIM #612621), typically inherited in an autosomal dominant manner. The ClinVar database reports numerous pathogenic variants distributed across the gene, most frequently associated with intellectual disability and related neurodevelopmental phenotypes. Reflecting the strength of genetic evidence, *SYNGAP1* is classified as a high-confidence syndromic ASD gene in the SFARI Gene database (score 1S). Consistent with this classification, constraint metrics from DECIPHER indicate strong intolerance to loss-of-function variants (pLI = 1; pLOF = 0.870), as well as marked intolerance to missense variation (Z score = 7.69), supporting a haploinsufficiency mechanism underlying disease pathogenesis.

Within the same mechanistic framework, *SHANK3* deficiency has been shown to induce autistic-like phenotypes through alterations extending beyond synaptic dysfunction to include oligodendrocyte abnormalities and disrupted intracellular signaling [155,156]. Experimental studies have demonstrated that loss of *SHANK3* leads to hyperactivation of the ERK branch of the MAPK pathway, which interferes with oligodendrocyte maturation and contributes to hypomyelination. Further evidence indicates that *SHANK3* deficiency is associated with myelin abnormalities in both the central and peripheral nervous systems, contributing to syndromic forms of ASD [157]. These findings suggest that MAPK dysregulation may affect not only neuronal signaling but also glial development and myelin formation, thereby influencing neural circuit connectivity in ASD. Transcriptomic analyses further revealed the concomitant dysregulation of WNT signaling, including the upregulation of Wnt5a, a key ligand of the non-canonical WNT pathway, in *SHANK3*-deficient oligodendrocytes [156]. The combined alterations in MAPK and WNT signaling highlight a convergence of intracellular pathways that regulate cytoskeletal dynamics, differentiation, and myelin-associated processes, providing a mechanistic link between *SHANK3* mutations, glial dysfunction, and ASD-related neurodevelopmental phenotypes.

**Figure 5:**
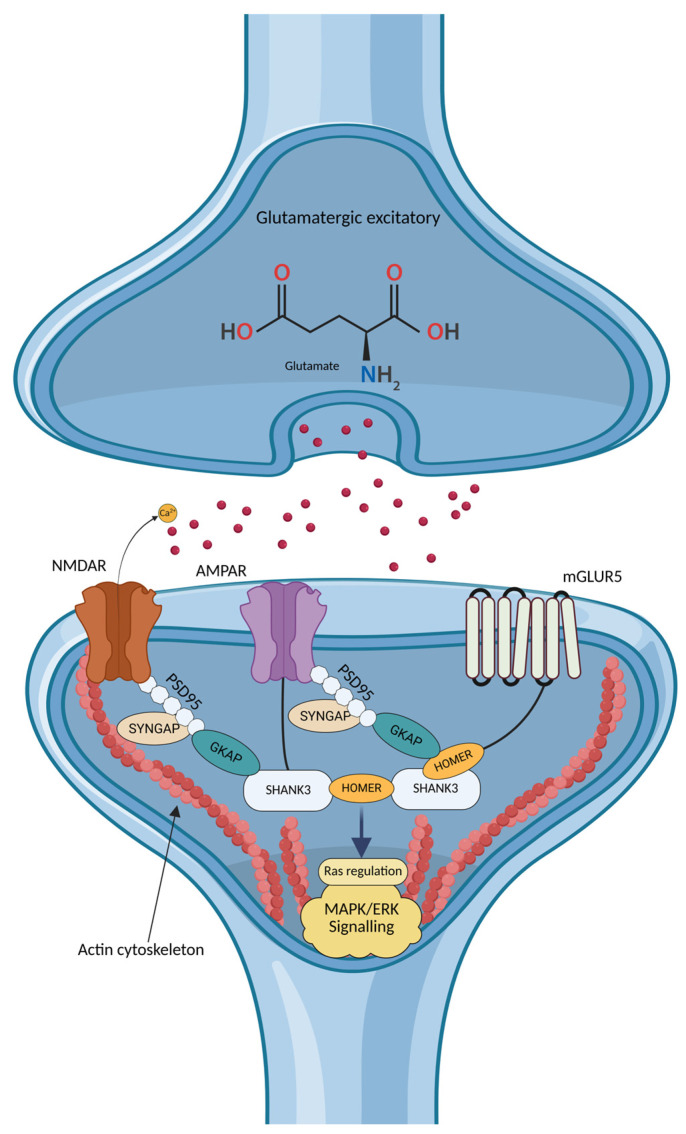
The glutamatergic postsynaptic density organization and convergence of ASD-associated scaffold proteins on Ras–MAPK/ERK signaling. Glutamate released from the presynaptic terminal activates ionotropic NMDA receptors (NMDAR) and metabotropic glutamate receptor 5 (mGluR5) located on the postsynaptic membrane. NMDAR-mediated Ca^2+^ influx and mGluR5 signaling initiate downstream pathways that converge on the Ras-dependent activation of the MAPK/ERK cascade. Within the postsynaptic density (PSD), scaffold proteins, including PSD95 (DLG4), GKAP, SHANK3, and HOMER, organize receptor complexes and anchor them to the actin cytoskeleton, thereby regulating the dendritic spine structure and synaptic stability. SYNGAP1 functions as a Ras GTPase-activating protein at PSD95–NMDAR complexes, modulating MAPK/ERK activation downstream of synaptic stimulation. Genetic alterations affecting these proteins disrupt excitatory synaptic signaling and intracellular pathways implicated in ASD, highlighting the convergence of glutamatergic neurotransmission and MAPK signaling in ASD pathophysiology.

In addition to its role in intracellular signaling, SHANK3 is tightly interconnected with glutamatergic neurotransmission through its function as a major postsynaptic density scaffolding protein [158]. SHANK3 organizes multiprotein complexes at excitatory synapses by linking NMDA-type and metabotropic glutamate receptors to the actin cytoskeleton via interactions with GKAP/PSD-95 and HOMER proteins, respectively. Mechanistically, the PDZ domain of SHANK3 binds to the C-terminus of guanylate kinase–associated protein (GKAP), which is associated with the PSD95–NMDA receptor complex, thereby stabilizing postsynaptic architecture and regulating synaptic signaling [159]. Disruption of these interactions compromises dendritic spine organization and contributes to glutamatergic dysfunction observed in ASD. Figure 5 illustrates the molecular architecture of an excitatory glutamatergic synapse and its coupling to intracellular Ras–MAPK/ERK signaling pathways.

Functional annotations further support the central role of SHANK3 in synaptic and intracellular signaling processes. Gene Ontology (GO) terms associated with SHANK3 include regulation of the MAPK cascade (GO:0000165), positive regulation of glutamatergic synaptic transmission (GO:0051968), and NMDA receptor clustering at the postsynaptic membrane (GO:0097114), highlighting its involvement at the interface between synaptic signaling and downstream intracellular pathways.

Experimental models reinforce these mechanistic insights. Complete loss of SHANK3 in mouse models disrupts mGluR5–Homer scaffolding complexes and alters corticostriatal connectivity, resulting in circuit-level abnormalities that recapitulate ASD-like behavioral phenotypes [160,161]. Consistent with these findings, zebrafish knockdown models of ASD-associated genes, including *SHANK3* and *SYNGAP1*, exhibit similar behavioral alterations, supporting a conserved role for postsynaptic scaffold proteins in neural circuit development and function [162].

Overall, these observations position SHANK3 as a key molecular hub linking glutamatergic signaling, MAPK-dependent intracellular pathways, and activity-dependent synaptic organization. Disruption of SHANK3-mediated scaffolding therefore represents a convergent mechanism through which diverse genetic alterations can impair synaptic plasticity and neural connectivity in ASD.

MAPK signaling is also closely interconnected with other pathways implicated in ASD, including WNT and PI3K–AKT–mTOR signaling [163]. Cross-talk between these cascades allows for the coordinated regulation of neuronal growth, dendritic arborization, and protein synthesis. For example, activation of NMDA receptors during excitatory synaptic transmission can trigger ERK phosphorylation, influencing local translation and gene expression programs that are essential for learning and memory. Dysregulation of these activity-dependent processes may contribute to excitation–inhibition imbalance, a core neurobiological feature of ASD.

Furthermore, significant cross-talk exists between the MAPK and calcium signaling pathways, reflecting the multisystem involvement observed in ASD [164,165]. The functional interaction between these signaling cascades contributes to shared molecular mechanisms across neurodevelopmental and psychiatric conditions, highlighting a convergence on MAPK- and calcium-dependent pathways. Beyond synaptic regulation, MAPK signaling influences neurotrophic factor responses, including brain-derived neurotrophic factor (BDNF) signaling, which has been linked to altered neuronal connectivity and plasticity in ASD [166,167]. Perturbations in MAPK-mediated transcriptional regulation may therefore result in widespread effects on neuronal development and network stability. Importantly, many ASD risk genes do not act within a single pathway but instead converge on shared intracellular signaling hubs, such as MAPK, supporting a model in which diverse genetic alterations disrupt common downstream mechanisms.

## 5. Conclusions

ASD represents a highly heterogeneous neurodevelopmental condition in which genetic, epigenetic, and environmental factors converge to shape the clinical presentation and disease trajectory. Advances in NGS technologies have markedly expanded our understanding of the genetic architecture of ASD, revealing a complex landscape composed of rare de novo variants, inherited mutations, and CNVs that contribute to variable neurodevelopmental phenotypes. Despite significant progress, a substantial proportion of individuals remain molecularly undiagnosed, underscoring the need for integrated genomic strategies and functional investigations to fully elucidate ASD etiology.

Emerging evidence highlights the convergence of diverse genetic alterations on shared molecular pathways, including synaptic signaling, WNT, mTOR, and MAPK cascades, which regulate neuronal differentiation, circuit formation, and activity-dependent plasticity. In particular, genes involved in glutamatergic and GABAergic neurotransmission emphasize the central role of excitation–inhibition balance in ASD pathophysiology. Disruption of postsynaptic scaffold proteins and signaling regulators further illustrates how synaptic dysfunction links molecular abnormalities to alterations in neural connectivity and behavior. These findings support a model in which ASD arises not from isolated genetic defects but from the perturbation of interconnected signaling networks that govern brain development and synaptic homeostasis.

From a clinical perspective, the integration of genomic data with phenotypic characterization remains essential for improving diagnostic yields and guiding genetic counselling. WES and WGS, complemented by curated databases and functional studies, are progressively refining the interpretation of variants of uncertain significance and enabling a more personalized approach to patient management. At the same time, ethical considerations surrounding data interpretation, incidental findings, and communication of genetic risk highlight the importance of multidisciplinary expertise in ASD clinical genetics.

Future studies should focus on expanding long-read sequencing technologies, improving functional validation pipelines, and integrating multi-omics approaches to better capture non-coding variation and regulatory mechanisms. Ultimately, a deeper understanding of convergent molecular pathways may facilitate the identification of novel therapeutic targets and promote the development of precision medicine strategies aimed at modulating synaptic signaling and restoring neural circuit balance in ASD. By bridging genomic discoveries with clinical application, the ongoing integration of molecular genetics into ASD research holds promise for advancing diagnosis, prognosis, and individualized care.

## Figures and Tables

**Table 1:** Overview and comparison of next-generation sequencing (NGS) approaches for genetic diagnosis of ASD.

Approach	Variant	Yield	Advantages	Limitations	References
Targeted gene panel	SNVs, small indels; limited CNVs	5–15%	High sequencing depth, low cost, fast turnaround, easier interpretation	Restricted to selected genes; unable to detect novel genes, non-coding variants, or complex structural rearrangements	[37,41]
WES	SNVs, small indels; limited CNVs	10–30%	Broad gene coverage; good balance between cost and diagnostic yield; enables novel gene discovery	Misses non-coding variants, deep intronic changes, and many structural variants	[27,28]
WGS	SNVs, indels, CNVs, SVs, regulatory and deep intronic variants	20–40%	Uniform coverage; improved detection of CNVs and non-coding variants; comprehensive variant discovery	Higher cost; increased data complexity; limited interpretability of non-coding variants	[35,36]
lrGS	SNVs, indels, CNVs, SVs, CGRs, repeat expansions, haplotype phasing	Not yet fully established	Superior resolution of complex genomic regions; accurate detection of SVs, CGRs, and repeat expansions	High cost; lower throughput; limited availability in routine clinical practice	No reference on diagnostic yield

WES: whole-exome sequencing, WGS: whole-genome sequencing; lrGS: long-read genome sequencing; SNV: single-nucleotide variant; CNV: copy number variant; SV: structural variant; CGR: complex genomic rearrangement.

**Table 2:** Recurrent copy number variants (CNVs) associated with ASD. Data from patients were retrieved from the DECIPHER database. The abbreviations used are explained in the footnotes.

CNV	Size	Neurodevelopmental Phenotype	Reference
1q21.1	Del 1.35 Mb	ID	[52]
	Dup 1.35 Mb	ASD, ID	[53]
3q29	Del 1.62 Mb	ID, FD	[54]
	Dup 1.62 Mb	Mental retardation, microcephaly	[55,56]
5q35	Dup 1.03 Mb	Aggressive behavior, ASD	Patients: 276387
	Dup 420.13 kb	ASD, GDD	Patients: 451406
7q11.23	Dup 1.40 Mb	DSLD, ID, SAS, ASD	[57]
	Del 1.40 Mb	ID, FD, SAS, SS	[58]
8p23.1	Del 3.66 Mb	Hyperactivity, ID	[59]
	Dup 3.66 Mb	FD, BA, DSLD, ID	[60]
15q13.3	Del 1.54 Mb	FD, ID, seizures	[61]
16p11.2	Dup 0.593 Mb	ASD, SCZ, GDD, BA, FD, epilepsy	[62,63,64]
	Del 8.69 Mb	FD, ID, ASD	[65]
16p12.2	Dup 7.81 Mb	FD, ASD, GDD, ID, microcephaly, SS	[66]
16p13.11	Del 1.50 Mb	ASD, epilepsy	[67]
	Dup 1.50 Mb	ASD, neurocognitive disease	[67,68]
16p13.3	Del 0.1551 Mb	N\A	[69]
17p11.2	Dup 3.45 Mb	ASD, hyperactivity, SAS, SS	[70]
	Del 3.45 Mb	ID, SS, SD, stereotypic behavior	[71]
17q11.2	Dup 0.312 Mb	ADHD, ASD, ID	Patients: 288818
17q12	Dup 1.47 Mb	ASD, DSLD, MD	Patients: 500957
	Del 1.74 Mb	ASD	Patients: 490194

ID: intellectual disability; ASD: autism spectrum disorder; FD: facial dysmorphia; GDD: global developmental delay; DSLD: delayed speech and language disorder; SAS: short attention span; SS: short stature; ADHD: attention deficit and hyperactivity disorder; BA: behavioral abnormality; MD: motor delay.

**Table 3:** List of monogenic disorders presenting autism spectrum disorder (ASD) in their clinical manifestations.

Syndrome	Gene	Localization	OMIM	Inheritance
Tuberous sclerosis complex	*TSC1*	9q34.13	Tuberous sclerosis-1 (MIM #191100)	AD
Tuberous sclerosis complex	*TSC2*	16p13.3	Tuberous sclerosis-2 (MIM #613254)	AD
Neurofibromatosis	*NF1*	17q11.2	Neurofibromatosis–Noonan syndrome (MIM #601321); neurofibromatosis, familial spinal (MIM #162210); neurofibromatosis, type 1 (MIM #162200); Watson syndrome (MIM #193520)	AD; AD; AD; AD
Neurofibromatosis	*NF2*	22q12.2	Schwannomatosis, vestibular (MIM #101000)	AD
Rett syndrome	*MECP2*	Xq28	Autism susceptibility (MIM #300496); encephalopathy, neonatal severe (MIM #300673); intellectual developmental disorder (MIM #300055); intellectual developmental disorder, Lubs type (MIM #300260); Rett syndrome (MIM #312750)	XL; XLR; XLR; XLR; XLD
Fragile X syndrome	*FMR1*	Xq27.3	Fragile X syndrome (MIM #300624); fragile X tremor/ataxia syndrome (MIM #300623); premature ovarian failure 1 (MIM #311360)	XLD; XLD; XL
Angelman syndrome	*UBE3A*	15q11.2	Angelman syndrome (MIM #105830)	AD

AD: autosomal dominant; XL: X-linked; XLD: X-linked dominant; XLR: X-linked recessive.

## Data Availability

No new data were created or analyzed in this study. Data sharing is not applicable to this article.

## Supplementary Materials

The following supporting information can be downloaded at: https://www.mdpi.com/article/10.3390/ijms27073278/s1.

## Abbreviations

The following abbreviations are used in this manuscript:

ACMG	American College of Medical Genetics and Genomics
AD	Autosomal dominant
ADHD	Attention deficit and hyperactivity disorder
ADI-R	Autism Diagnostic Interview Revised
ADOS	Autism Diagnostic Observation Schedule
AR	Autosomal recessive
ASD	Autism spectrum disorder
BD	Bipolar disorder
BDNF	Brain-derived neurotrophic factor
CGR	Complex genomic rearrangement
CHOOSE	CRISPR–human organoids–single-cell RNA sequencing
CMA	Chromosomal microarray analysis
CNV	Copy number variant
DAT	Dopamine transporter
DSM-5	*Diagnostic and Statistical Manual of Mental Disorders, Fifth Edition*
EAAT	Excitatory amino acid transporters
GWAS	Genome-wide association studies
ID	Intellectual disability
lrGS	Long-read genome sequencing
MAPK	Mitogen-activated protein kinase
NDD	Neurodevelopmental disorder
NGS	Next-generation sequencing
NVIQ	Nonverbal Intelligence Quotient
OCB	Obsessive–compulsive disorder
OMIM	Online Mendelian Inheritance in Man
SCZ	Schizophrenia
SNV	Single-nucleotide variant
SFARI	Simons Foundation Autism Research Initiative
srGS	Short-read genome sequencing
SV	Structural variant
VGLUT	Vesicular loading through
WES	Whole-exome sequencing
WGS	Whole-genome sequencing
